# On the measurement errors in SAR supervision introduced by directional couplers

**DOI:** 10.1007/s10334-025-01287-7

**Published:** 2025-08-04

**Authors:** Stephan Orzada, Thomas M. Fiedler, Jan Kesting, Max Joris Hubmann, Mark E. Ladd

**Affiliations:** 1 Medical Physics in Radiology, German Cancer Research Center (DKFZ), Im Neuenheimer Feld 280, 69120 Heidelberg, Germany; 2 Research Campus STIMULATE, Magdeburg, Germany; 3 Faculty of Electrical Engineering and Information Technology, Otto‐von‐Guericke University, Magdeburg, Germany; 4 Erwin L. Hahn Institute for MRI, University Duisburg-Essen, Essen, Germany; 5 Faculty of Physics and Astronomy, Heidelberg University, Im Neuenheimer Feld 226, 69120 Heidelberg, Germany; 6 Faculty of Medicine, Heidelberg University, Im Neuenheimer Feld 672, 69120 Heidelberg, Germany

**Keywords:** SAR, Directional couplers, Measurement error, MRI, Virtual observation points, pTx

## Abstract

**Introduction:**

This study proposes a framework for determining the calculation error in online SAR supervision introduced by directional couplers.

**Materials and methods:**

A mathematical framework is introduced showing how the error in the measured excitation vector compared to the actual excitation vector can be rewritten as a new set of virtual observation points (VOPs). By comparing the new set of VOPs to the original VOPs through an optimization, the maximum underestimation of SAR can be calculated. The framework is then applied to five different RF arrays.

**Results:**

The results show that the error in SAR calculation is very dependent on the position of the reference plane of the directional coupler measurements and the S-parameters of the array. To have an error of less than 5%, directional couplers with a directivity better than 40 dB are necessary for the worst case of the investigated arrays.

**Discussion:**

The framework presented in this paper provides an approach for calculating a safety factor to account for the inaccuracies introduced by directional coupler measurements in online SAR supervision. The framework can also be adapted to other types of measurements.

## Introduction

To excite the spins in a magnetic resonance imaging experiment, radiofrequency (RF) fields at the Larmor frequency of the corresponding nuclei are applied. When these fields interact with lossy body tissues, RF power is absorbed, resulting in heating of the tissue. To ensure patient safety [1], MR systems monitor the RF power during the examination and calculate the specific absorption rate (SAR) in W/kg with information about the electric field distribution from a simulation model: $\mathrm{SAR}\left(r\right)=\frac{\Delta {P}_{\mathrm{abs}}}{\rho \Delta V}=\frac{\sigma \left(r\right)}{2\rho \left(r\right)}\frac{1}{T}\underset{0}{\overset{T}{\int }}{\Vert E\left(t,r\right)\Vert }^{2}dt$ [2]. The necessary power measurements can be performed using directional couplers (DiCos) placed in the transmit path [3–5] or sniffer coils placed on the transmit coil [6].

For single-channel systems, which are the clinical standard, the SAR supervision is straightforward, as the SAR scales linearly with forward RF power. It is sufficient to measure the amplitude of the power, disregarding the phase information. In multi-channel transmit systems, SAR is a function of the complex excitation vector containing the amplitude and phase for each channel. In this case, the RF fields for each channel in the simulation are normalized to the corresponding forward signal. These normalized fields can then be multiplied by the forward signals from the online supervision and superimposed to calculate the actual field. Since it is computationally very demanding to do this for every point in the simulation domain, the virtual observation points formalism introduced by Eichfelder et al. [7] can be used to simplify the calculation.

There are several sources of error contributing to the overall error in the calculation of SAR, which include simulation model inaccuracies (e.g., body models are not identical to the subject), receiver noise and digitization errors, as well as errors from the directional couplers due to their finite directivity. To account for these errors to not underestimate SAR, safety factors need to be included in the SAR calculation from the measured values of the online supervision system. These safety factors are estimated for the specific hardware.

In practice, using directional couplers to measure the forward signal introduces measurement errors due to their finite directivity. While the potential error in single-channel systems is easy to estimate, the impact of directional coupler measurement errors on SAR in multi-channel transmit systems is more complicated to characterize. In this work, we present a comprehensive method to calculate the measurement error introduced by directional couplers in multi-channel systems based on virtual observation points [7] and the convex optimization (CO) criterion presented by Gras et al. [8]. The intention is to provide a framework to exactly determine the error on the SAR calculation to allow for the calculation of safety factors.

## Materials and methods

### Forward and reflected signals

**Fig. 1:**
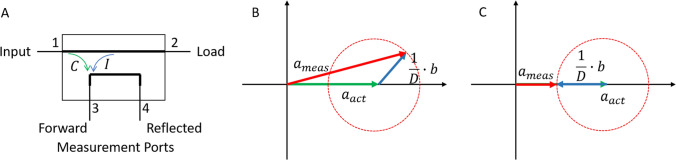
**A** Simplified schematic of a directional coupler. **B** Vector representation of measured signals at a coupled port of a directional coupler. **C** Worst-case phase, where the measured signal ${a}_{\mathrm{meas}}$ is minimal because ${a}_{\mathrm{act}}$ and $b$ have opposed phase

A simplified example of a directional coupler [9] is shown in Fig. 1A. The signal is transmitted from Port 1 (Input) to Port 2 (Load, e.g., antenna). A portion of the signal entering Port 1 is coupled to Port 3 with a coupling coefficient $C$ and a smaller part from Port 2 to Port 3 with an isolation coefficient $I$. The ratio between these two coefficients is called directivity $D$. An ideal directional coupler would have an isolation coefficient $I$ of 0 and therefore an infinite directivity.

Due to the finite directivity of real-world directional couplers, the measured complex forward voltage signal ${a}_{\mathrm{meas}}$ consists of the summation of the signal we would ideally measure ${a}_{act}$ and the reflected signal $b$ weighted with the inverse of the directivity $D$:

1 $${a}_{\mathrm{meas}}={a}_{\mathrm{act}}+\frac{1}{D}\bullet b$$

Since ${a}_{\mathrm{act}}$ and $b$ are vector representations of complex signals, the measured signal also depends on the phase between $b$ and ${a}_{\mathrm{act}}$. Therefore, the result for ${a}_{\mathrm{meas}}$ for a certain amplitude of $b$ is somewhere on the dashed circle in Fig. 1B.

The worst-case error, meaning the largest underestimation of SAR, for a measurement with a directional coupler and a single transmitter is shown in Fig. 1C, where ${a}_{\mathrm{act}}$ and $b$ are 180° out of phase. In this case, the measured forward signal is smaller than the actual forward signal, leading to an underestimation of SAR. Since the maximum possible reflected power in a single-channel system is identical to the applied forward power and the directivity is known, the worst-case underestimation can be calculated directly and compensated for.

For parallel transmit systems, the calculation of a worst case is much more difficult. First, the SAR not only depends on the amplitude of the forward signal, but also on its phase and is therefore a function of the complex excitation vector ${\boldsymbol{x}}$, whose number of elements is equal to the number of channels. Here, each complex entry in the excitation vector corresponds to a complex signal ${a}_{act}$ as introduced above. Second, the total reflected power in a channel is a function not only of the forward power in this channel, as in a single-channel system, but also depends on the amplitudes and phases of the signals from the other channels coupling into this channel. The latter contributions can be significantly higher than the channel’s own internal reflection [10].

In practice, the excitation vector $\mathbf{x}$ containing the complex signals for all channels is measured using directional couplers between the amplifiers and the RF array. Due to the finite directivity $D$ of the directional couplers, the measurement yields the values

2 $${{\boldsymbol{x}}}_{\mathrm{meas}}=\frac{1}{D}\bullet {\boldsymbol{y}}+{{\boldsymbol{x}}}_{\mathrm{act}}$$

where $\mathbf{y}$ is the complex vector of the reflected signals. Each complex entry of ${{\boldsymbol{x}}}_{\mathrm{meas}}$ now corresponds to the respective ${a}_{\mathrm{meas}}$ of the channels.

With the complex S-parameter matrix $\mathbf{S}$ of an RF array [11] and the complex excitation vector ${{\boldsymbol{x}}}_{\mathrm{act}}$ of a multi-channel system, the vector of the complex reflected signal $\mathbf{y}$ can be calculated as

3 $${\boldsymbol{y}}=\frac{1}{\mu }{e}^{i\phi }\mathbf{S}{{\boldsymbol{x}}}_{\mathrm{act}}$$

Here, the term ${e}^{i\phi }$ describes the potential phase difference between the planes of reference of the S-parameters (for example from a simulation) and the directional coupler measurement. We use a single phase for all channels, as it is a reasonable assumption that cable length is close to identical. Furthermore, $\frac{1}{\mu }$ describes the extra attenuation introduced by the cable between the points of S-parameter measurement and directional coupler measurement. With these two terms, we can effectively shift the measurement of the forward power along the transmit cable. This is necessary as our results show that the exact position of measuring the forward power is important for the error.

With Eq. 3, the measured excitation vector can be written as

4 $${{\boldsymbol{x}}}_{\mathrm{meas}}={\frac{1}{\mu }e}^{i\phi }\frac{1}{D}\bullet \mathbf{S}{{\boldsymbol{x}}}_{\mathrm{act}}+{{\boldsymbol{x}}}_{\mathrm{act}}$$

Generally, the directional couplers are placed as close to the coils as possible. As the attenuation over a length of less than one wavelength is fairly low in the transmit cables of an MRI system and therefore negligible compared to the directivity and is in any case equivalent to a slight increase in directivity, we disregard it in the further steps.

### SAR calculation

As stated in the introduction, to calculate the local SAR in the online supervision of a multi-channel MRI system, often so-called virtual observation points (VOPs) are used [7] that are derived from Q-matrices [12]. With the VOP matrices ${\mathbf{Q}}_{{\boldsymbol{j}}}$ of the VOP set ${\mathcal{Q}}_{\mathrm{act}}$ and the complex excitation vector ${\boldsymbol{x}}$, SAR in the volume of the virtual observation point $j$ can be easily calculated as

5 $${\mathrm{SAR}}_{j}={{\boldsymbol{x}}}^{H}{\mathbf{Q}}_{{\boldsymbol{j}}}{\boldsymbol{x}}$$

Since the measured excitation vector is different from the actual excitation vector, the SAR calculations yield different results

6 $${\mathrm{SAR}}_{j,\mathrm{meas}}={{\boldsymbol{x}}}_{\mathrm{meas}}^{H}{\mathbf{Q}}_{j}{{\boldsymbol{x}}}_{\mathrm{meas}}={{\left({e}^{i\phi }\frac{1}{D}\bullet \mathbf{S}{{\boldsymbol{x}}}_{\mathrm{act}}+{{\boldsymbol{x}}}_{\mathrm{act}}\right)}^{H}\mathbf{Q}}_{j}\left({e}^{i\phi }\frac{1}{D}\bullet \mathbf{S}{{\boldsymbol{x}}}_{\mathrm{act}}+{{\boldsymbol{x}}}_{\mathrm{act}}\right)$$

In this form, it is not easy to compare the SAR calculated from the measurements to the actual SAR.

### Error calculation

Using a few simple steps, Eq. 6 can be reformulated as follows:

7 $$\begin{aligned} {\mathrm{SAR}}_{{j,{\mathrm{meas}}}} = & \left( {e^{i\phi } \frac{1}{D} \cdot {\mathbf{S}}{\boldsymbol{x}}_{{{\mathrm{act}}}} + {\boldsymbol{x}}_{{{\mathrm{act}}}} } \right)^{H} {\mathbf{Q}}_{j} \left( {e^{i\phi } \frac{1}{D} \cdot {\mathbf{S}}{\boldsymbol{x}}_{{{\mathrm{act}}}} + {\boldsymbol{x}}_{{{\mathrm{act}}}} } \right) \\ = & {\boldsymbol{x}}_{{{\mathrm{act}}}}^{H} \left( {e^{i\phi } \frac{1}{D} \cdot {\mathbf{S}} + {\mathbf{I}}} \right)^{H} {\mathbf{Q}}_{j} \left( {e^{i\phi } \frac{1}{D} \cdot {\mathbf{S}} + {\mathbf{I}}} \right){\boldsymbol{x}}_{{{\mathrm{act}}}} \\ \user2{ } = & {\boldsymbol{x}}_{{{\mathrm{act}}}}^{H} {\mathbf{M}}_{{{\mathrm{error}}}}^{H} {\mathbf{Q}}_{j} {\mathbf{M}}_{{{\mathrm{error}}}} {\boldsymbol{x}}_{{{\mathrm{act}}}} \\ = & {\boldsymbol{x}}_{{{\mathrm{act}}}}^{H} {\tilde{\mathbf{Q}}}_{{j,{\mathrm{meas}}}} {\boldsymbol{x}}_{{{\mathrm{act}}}} \\ \end{aligned}$$

Here, $\mathbf{I}$ is the identity matrix and we designate ${\mathbf{M}}_{\mathrm{error}}$ the error matrix. Through the above reformulation, we now have a new set ${\mathcal{Q}}_{meas}$ of VOP matrices ${\widetilde{\mathbf{Q}}}_{j,\mathrm{meas}}$.

In the following, we use $k$ instead of *j* as the running index to avoid confusion with the running index $j$ of the original set of matrices ${\mathcal{Q}}_{\mathrm{act}}$; nevertheless, a matrix ${\widetilde{\mathbf{Q}}}_{k,\mathrm{meas}}$ is derived from ${\mathbf{Q}}_{j}$ if $k=j$. We can easily compare ${\widetilde{\mathbf{Q}}}_{k,\mathrm{meas}}$ of ${\mathcal{Q}}_{\mathrm{meas}}$ to the actual SAR matrices ${\mathbf{Q}}_{j}$ of ${\mathcal{Q}}_{\mathrm{act}}$ for all possible values of ${\boldsymbol{x}}\in {\mathbb{C}}^{{N}_{c}}$ using the CO criterion introduced by Gras et al. [8]: to find the maximum relative error $r$ introduced by the measurement with directional couplers, we now need to solve

8 $$r\left(\phi \right)=\underset{{\mathbf{Q}}_{j}\in {\mathcal{Q}}_{\mathrm{act}}}{\mathrm{max}}\underset{{\boldsymbol{x}}\in {\mathbb{C}}^{{N}_{c}}}{\mathrm{max}}\left({\left(\underset{{\widetilde{\mathbf{Q}}}_{k,\mathrm{meas}}\in {\mathcal{Q}}_{\mathrm{meas}}}{\mathrm{max}}\left({{\boldsymbol{x}}}^{H}\left({\widetilde{\mathbf{Q}}}_{k,\mathrm{meas}}\right){\boldsymbol{x}}\right)\right)}^{-1}{{\boldsymbol{x}}}^{H}{\mathbf{Q}}_{j}{\boldsymbol{x}}\right)$$

The maximization over $\mathbf{x}$ can be reformulated as

9 $$r_{{{\mathrm{CO}}}} \left( {{\mathbf{Q}}_{j} ,\phi } \right) = \mathop {\max }\limits_{{{\boldsymbol{x}} \in {\mathbb{R}}^{{2N_{c} }} }} \left( {{\boldsymbol{x}}^{T} {\mathbf{Q}}_{{j,{\mathbb{R}}}} {\boldsymbol{x}}} \right)\;{\text{subject }}\;{\mathrm{to}}\;{\boldsymbol{x}}^{T} {\tilde{\mathbf{Q}}}_{{k,{\mathrm{meas}},{\mathbb{R}}}} {\boldsymbol{x}} \le 1,\user2{ }\forall {\tilde{\mathbf{Q}}}_{{k,{\mathrm{meas}}}} \in {\mathcal{Q}}_{{{\mathrm{meas}}}}$$

with

10 $${\mathbf{Q}}_{{j,{\mathbb{R}}}} = \left( {\begin{array}{*{20}c} {{\mathcal{R}}\left( {{\mathbf{Q}}_{j} } \right)} & { - {\Im }\left( {{\mathbf{Q}}_{j} } \right)} \\ {{\Im }\left( {{\mathbf{Q}}_{j} } \right)} & {{\mathcal{R}}\left( {{\mathbf{Q}}_{j} } \right)} \\ \end{array} } \right)\;{\mathrm{and}}\;{\tilde{\mathbf{Q}}}_{{k,{\mathrm{meas}},{\mathbb{R}}}} = \left( {\begin{array}{*{20}c} {{\mathcal{R}}\left( {{\tilde{\mathbf{Q}}}_{{k,{\mathrm{meas}}}} } \right)} & { - {\Im }\left( {{\tilde{\mathbf{Q}}}_{{k,{\mathrm{meas}}}} } \right)} \\ {{\Im }\left( {{\tilde{\mathbf{Q}}}_{{k,{\mathrm{meas}}}} } \right)} & {{\mathcal{R}}\left( {{\tilde{\mathbf{Q}}}_{{k,{\mathrm{meas}}}} } \right)} \\ \end{array} } \right)$$

Gras et al. showed in their paper that this is a convex problem [8]. The maximum over ${\mathbf{Q}}_{j}\in {\mathcal{Q}}_{\mathrm{act}}$ can be calculated by running the inner optimization for each element of ${\mathcal{Q}}_{\mathrm{act}}$ and choosing the maximum.

Since finding the maximum error over the phase difference between the reference planes of the S-parameter measurements and the directional coupler measurements is not a convex problem as our results will show, we run the phase difference $\phi$ from 0 to 2π in 360 steps.

### Numerical examples with five arrays

**Fig. 2:**
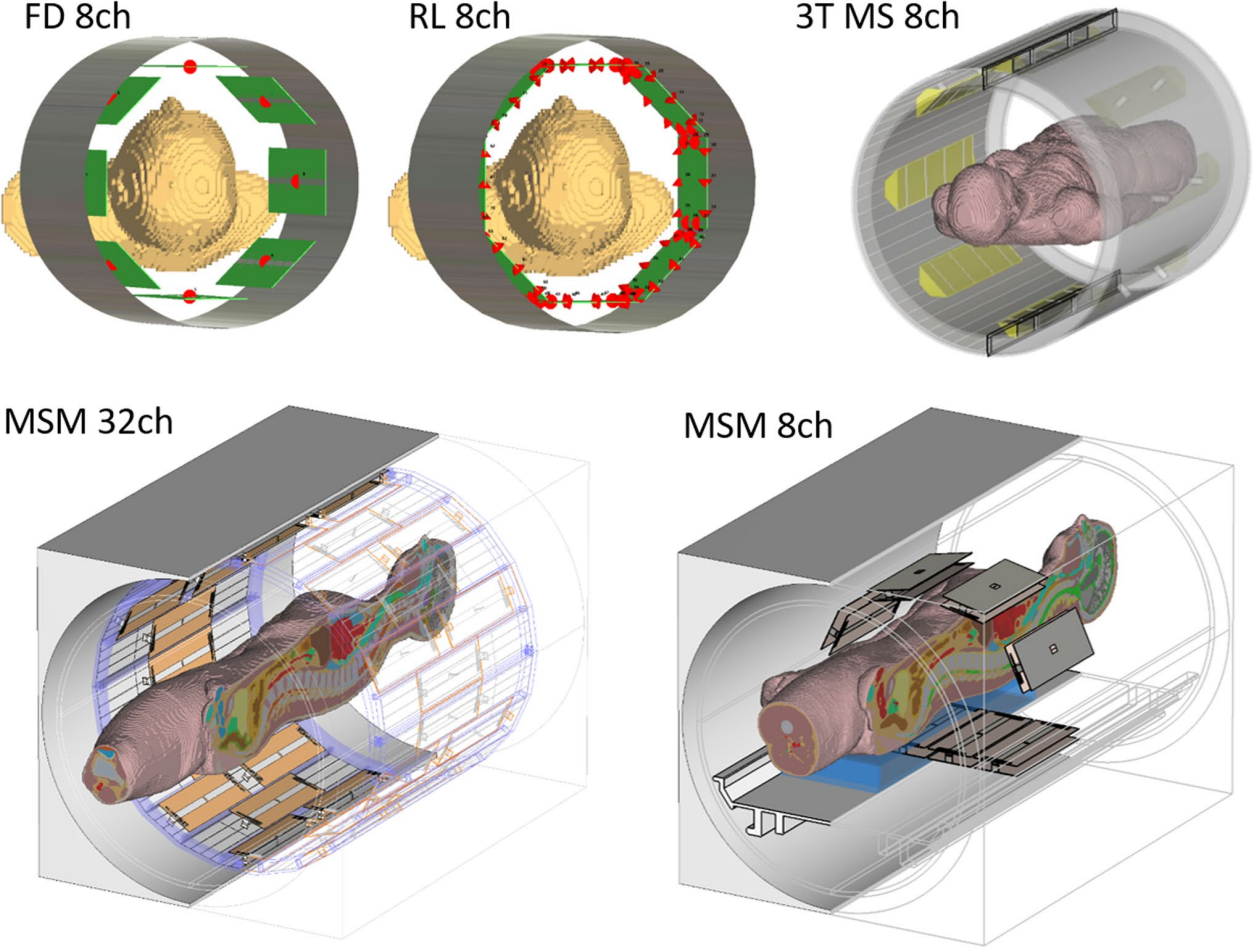
Coil array models used in this work. FD 8ch is a large head array consisting of 8 fractionated dipoles, RL 8ch is a large head array consisting of rectangular loops, both for 7 Tesla. 3T MS 8ch is an 8-channel body array for 3 Tesla consisting of 8 microstrip lines. MSM 32 is an integrated body array for 7 Tesla consisting of 32 microstrip lines with meanders, while MSM 8ch is local body array consisting of the same element type

To provide an impression on how large the error can be for different arrays and how much it varies, we used simulation data of five different RF arrays with realistic human body models from CST Microwave Studio (Dassault Systèmes, Vélizy-Villacoublay, France). Four of these are 7 T arrays: a fractionated dipole [13] head array with eight channels (FD 8ch), a rectangular loop head array with eight channels (RL 8ch), a 32-channel integrated body array of microstrip lines with meanders [14] (MSM 32ch), and an 8-channel flexible body array of microstrip lines with meanders [15] (MSM 8ch). Furthermore, a microstrip line body array for 3 T (3T MS 8ch) very similar to the work of Vernickel et al. [16] was simulated. Images of the CAD models are shown in Fig. 2. The SAR matrices were exported to Matlab and compressed using hybrid compression algorithms [17]. S-parameters were exported to calculate the error matrices. Since it is clear from Eq. 5 that the S-parameter matrix plays an important role for the extent of the error in the measurement, the eigenvalues of ${\mathbf{S}}^{H}\mathbf{S}$ were calculated according to the work of Kazemivalipour et al. [10]. This provides information on the total reflected power, with the highest eigenvalue providing the worst-case total reflected power. The eigenvalues can be used as a quality measure for the coupling of an array.

Two types of errors were investigated: 1) the maximum underestimation of local SAR was calculated as explained above, and 2) the maximum underestimation in the measurement of total forward power was evaluated by calculating one over the minimum eigenvalue of ${{{\mathbf{M}}_{\mathrm{sq}}=\mathbf{M}}_{\mathrm{error}}}^{H}{\mathbf{M}}_{\mathrm{error}}$. The second calculation is identical to using an identity matrix instead of a set of VOPs for the calculation of $r$.

All calculations were performed in Matlab R2023a (The MathWorks, Natick, MA, USA).

## Results

**Fig. 3:**
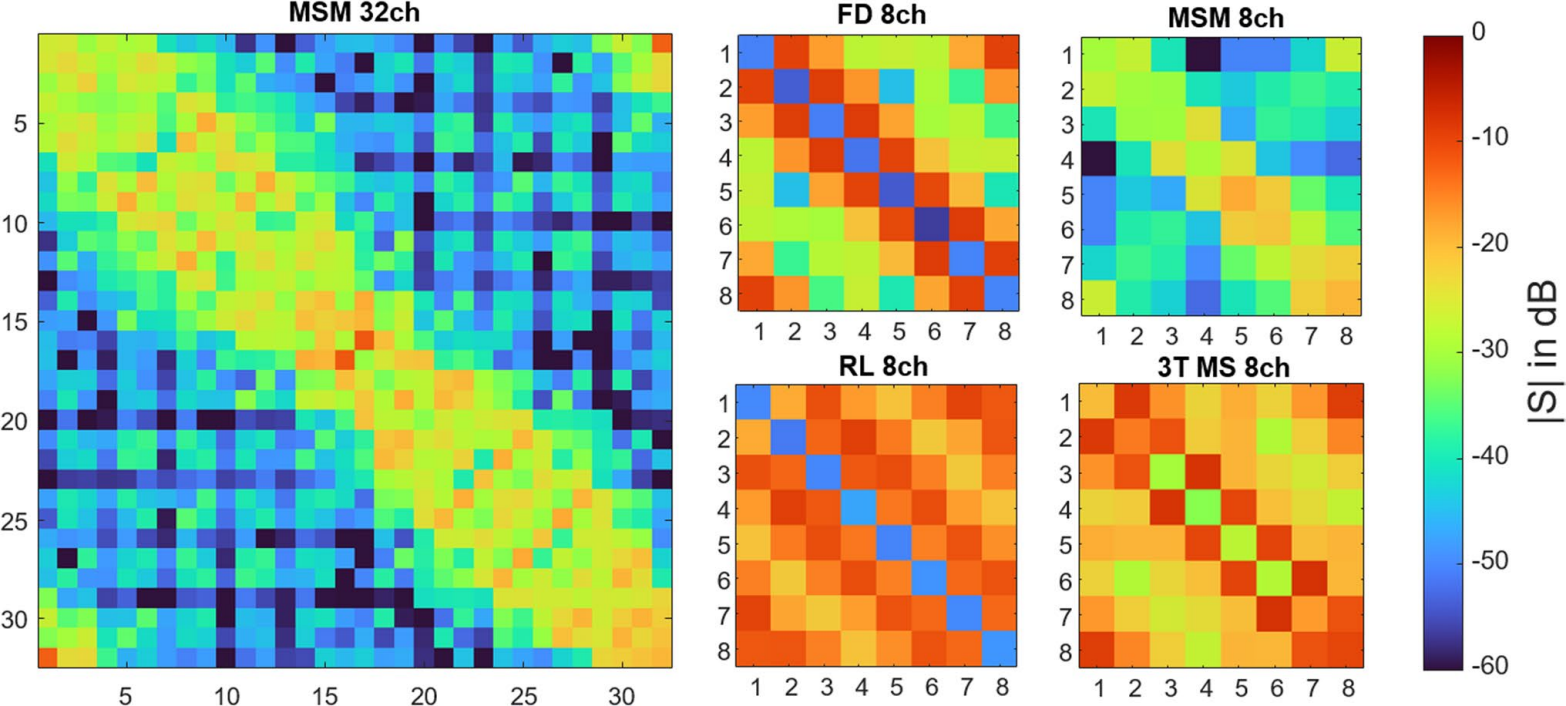
S-parameter matrices of all arrays (see Fig. 2). While the two arrays with microstrip line with meanders (MSM) elements show low coupling between elements, the other three arrays show rather high coupling

The S-parameters of the arrays are shown in Fig. 3. As can be expected from such a diverse set of arrays, the S-parameters are very different. Especially the MSM 8ch array shows very little coupling, since the coil elements used are intrinsically well decoupled and heavily loaded as they are close to the body. The three large-diameter 8-channel arrays exhibit strong coupling, as the FD 8ch array does not have any decoupling networks, the RL 8ch only uses capacitive nearest neighbor decoupling, and the decoupling network of the 3 T array is not very efficient.

**Fig. 4:**
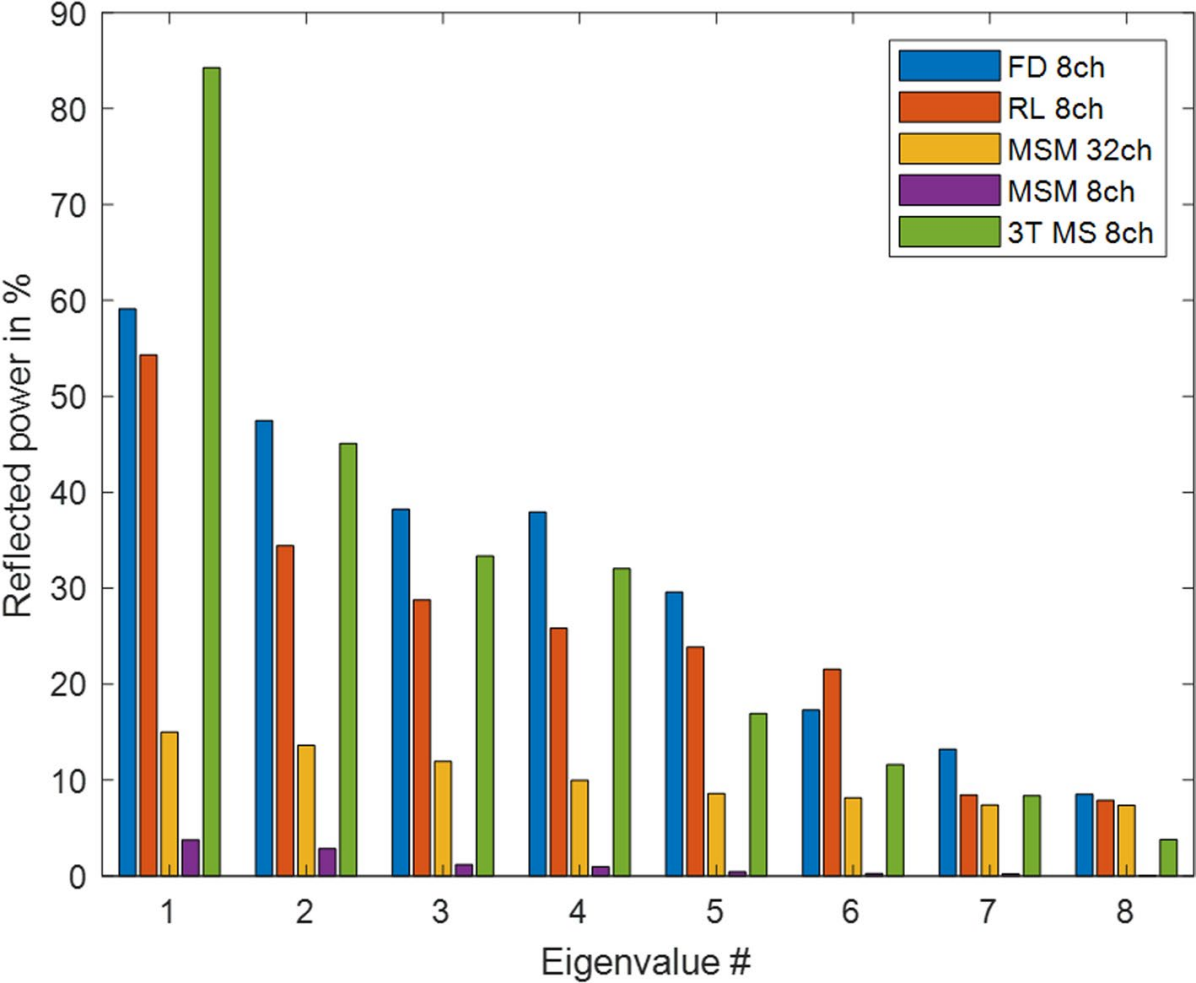
Reflected power corresponding to the eigenvalues of ${\mathbf{S}}^{H}\mathbf{S}$. For the 32-channel array, only the eight highest eigenvalues are shown for convenience

The differences in coupling are also shown in the eigenvalues of the square of the S-parameter matrix. Figure 4 gives the eight highest respective eigenvalues for the five arrays. The 3 T microstrip line array exhibits strongest overall reflection with 84% in the worst case. The two head arrays FD 8ch and RL 8ch also have modes with high reflected power. The two MSM arrays show the overall lowest reflections.

**Fig. 5:**
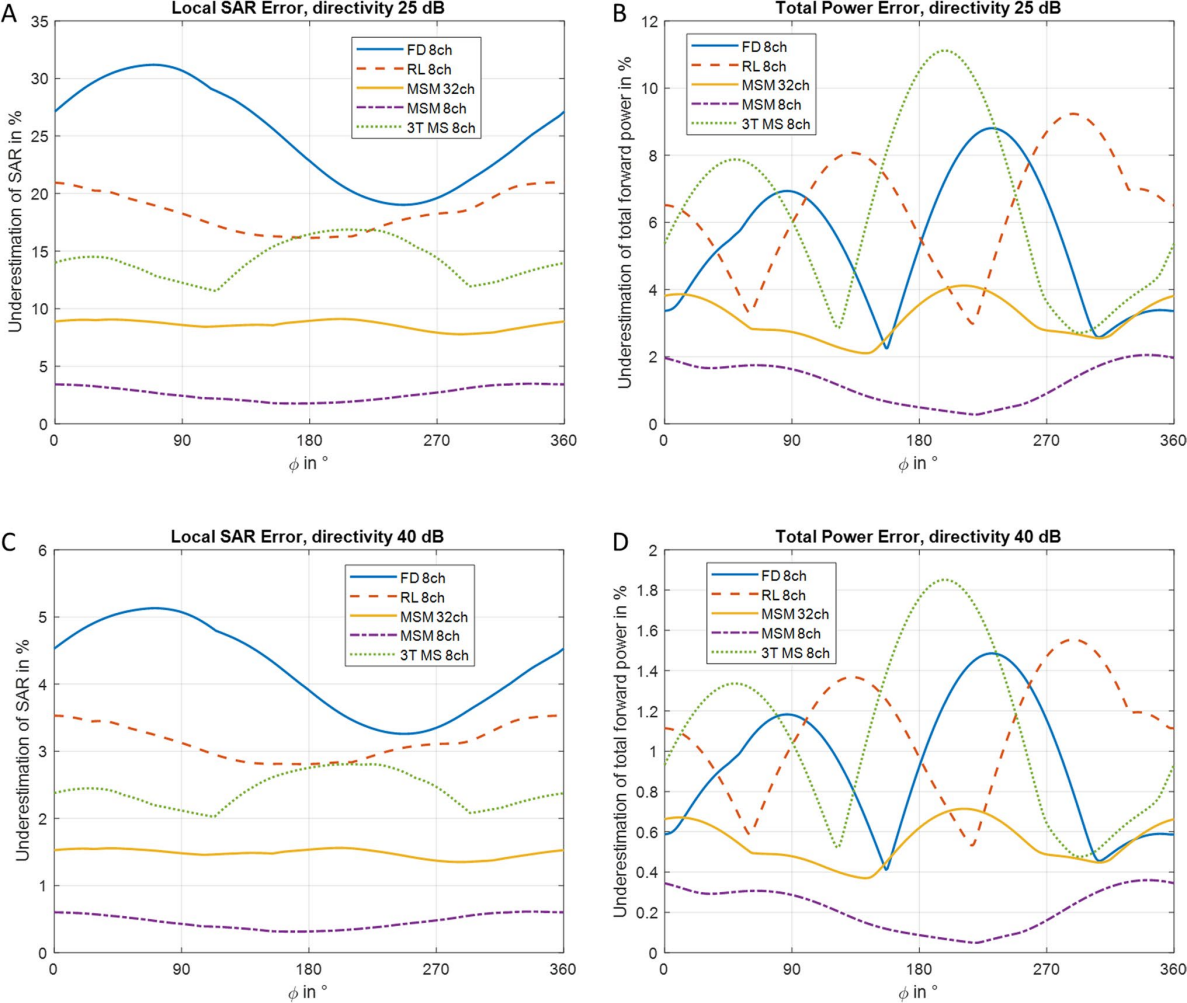
**A** Underestimation of local SAR in percent of the actual SAR over the phase difference between the plane of reference of the S-parameter measurement and the plane of reference of the directional coupler measurements for directional couplers with 25 dB directivity. **B** Underestimation of total forward power over the phase difference between the plane of reference of the S-parameter measurement and the plane of reference of the directional coupler measurements for directional couplers with 25 dB directivity. **C** Underestimation of local SAR for 40dB directivity. **D** Underestimation of total power for 40dB directivity

Figure 5A shows how the coupling translates to SAR calculation error when using directional couplers with 25 dB directivity, which is a common value for mid-class commercial directional couplers. As the SAR error can have more than one minimum, it is obvious that the optimization problem over $\phi$ is not convex. Depending on the angle $\phi$, the underestimation of local SAR can be 31.2% in the case of the FD 8ch array. The two MSM arrays only show 9.1% and 3.5% error for the 32-channel and 8-channel arrays, respectively. When looking at the error in total power measurement in Fig. 5B, one can see that the error is smaller than the error in the SAR calculation. As one would expect from the eigenvalue calculation, the 3 T array shows the largest error at about 11.1%, while the MSM 8ch array shows the smallest error with a maximum of 2.1%.

Since 25 dB is a good, but not an excellent, value for directivity, we also calculated the errors for a directivity of 40 dB (Fig. 5C and 5D), which can be achieved by a directivity tuner [18], for example. The curves are similar to those shown in Fig. 5, but with overall lower errors. The maximum SAR error for the FD 8ch array is 5.1%, the maximum power error is 1.9% for the 3T MS 8ch array.

## Discussion

The results show that the error in local SAR calculation when using measurement with directional couplers not only depends on the directivity of the directional couplers used, but also on the S-parameters and the structure of the SAR matrices that play an important role in the measurement error. The role of the S-parameters is a direct consequence of Eq. 5. If the array is ideally matched and decoupled, $\mathbf{S}$ would be a zero matrix and ${\mathbf{x}}_{meas}$ would be identical to ${\mathbf{x}}_{act}$. Low coupling in an array is important for power efficiency and the degrees of freedom in pTx experiments [19]. In this work, we show that it also reduces the error in SAR supervision when using directional couplers. Our results also show that a simple analysis of the error in total forward power is insufficient, as this error is smaller than the error in SAR calculation. Furthermore, in a comparison between arrays, an array with a higher error in forward power measurement does not necessarily also show a higher error in SAR calculation (cf. Figure 5).

The analysis in this paper implicates an unintuitive fact. The error in calculated local SAR becomes smaller if the directional couplers are further away from the array and closer to the amplifiers. The reason for this is that the cable attenuation will reduce the reflected power, while the forward power is increased. This corresponds to multiplying the S-parameter matrix with the square of the attenuation. On the other hand, moving the directional couplers away from the array reduces the fidelity of reflection measurements to check, for example, whether the S-parameters of an array are within specifications. Since the reflected power is generally lower than the forward power, it is favorable to position the directional couplers close to the array.

The presented framework for calculating the error can also be used for other methods of SAR supervision. In the case of sniffer coils [6] to measure the current on each transmit coil, the error matrix ${\mathbf{M}}_{\mathrm{error}}$ would be an identity matrix if each sniffer coil only collects signal from its respective coil element. Since in reality they would also collect signals from other coil elements, the other positions in the error matrix are filled with the complex correlation factors for the respective coils. A low correlation between channels corresponds to a high directivity for the directional coupler measurement.

It should be noted that finite directivity is not the only source of error in measurement of forward signals in MRI safety supervision, and that other error sources like digitization errors and noise need to be considered as well.

In conclusion, we present a framework for calculating the error in SAR supervision introduced by directional couplers that can also potentially be used for calculating the error for other means of measuring the per-channel transmission. The SAR measurement is better with directional couplers with higher directivity, but it is also dependent on the inter-element coupling of the array, emphasizing the value of well decoupled transmit elements.

## Data Availability

The code and the raw data for this work can be found at https://github.com/sOrzada/DiCo-SAR-Error.
